# Role of angiotensin-converting enzyme 2 in neurodegenerative diseases during the COVID-19 pandemic

**DOI:** 10.18632/aging.103993

**Published:** 2020-11-10

**Authors:** Zhenyu Li, Xiaolin Xu, Meiling Yang, Jianping Feng, Cunming Liu, Chun Yang

**Affiliations:** 1 Department of Anesthesiology and Perioperative Medicine, The First Affiliated Hospital of Nanjing Medical University, Nanjing 210029, China; 2 Department of Anesthesiology, Tongji Hospital, Tongji Medical College, Wuhan 430030, China

**Keywords:** angiotensin-converting enzyme 2, neurodegenerative diseases, the elderly, COVID-19

## Abstract

SARS-CoV-2 (severe acute respiratory syndrome coronavirus 2) uses the angiotensin-converting enzyme 2 (ACE2) receptor for infecting and spreading in humans. Studies have shown that the widespread expression of ACE2 in human tissues may be associated with organ function damage (e.g., lung, kidney, and stomach) in patients with coronavirus disease 2019 (COVID-19). However, in neurodegenerative diseases, whose pathogenesis is closely related to advanced age, ACE2 plays a neurotrophic and protective role by activating the ACE2/Ang-(1-7)/Mas axis, thus inhibiting cognitive impairment. Early reports have revealed that the elderly are more susceptible to COVID-19 and that elderly patients with COVID-19 have faster disease progression and higher mortality. Therefore, during the COVID-19 pandemic, it is crucial to understand the role of ACE2 in neurodegenerative diseases. In this paper, we review the relationship between COVID-19, neurodegenerative diseases, and ACE2, as well as provide recommendations for the protection of elderly patients with neurodegenerative diseases during the COVID-19 pandemic.

## INTRODUCTION

The global community is currently in the prevention and control phase of coronavirus disease 2019 (COVID-19). The International Committee on Taxonomy of Viruses named the virus causing COVID-19 as severe acute respiratory syndrome coronavirus 2 (SARS-CoV-2). From December 2019, the COVID-19 outbreak has affected nearly 20 million individuals worldwide. The World Health Organization specifically classified as a “pandemic” [1].

Currently, the median age of COVID-19 patients is approximately 50 years old, with the majority being males. Most patients experience mild symptoms, whereas approximately 25% of patients have a severe course requiring intensive care [2]. By contrast, adolescents and children usually experience mild symptoms [2]. Although SARS-CoV-2 leads to minor or mild flu-like symptoms in the majority of affected patients, it may cause severe, lethal complications, such as progressive pneumonia, acute respiratory distress syndrome, and organ failure, driven by inflammation and a cytokine storm syndrome [3]. The Case Fatality Rate (CFR) of COVID-19 varies greatly between regions and age groups.

The COVID-19 is associated with a high risk of morbidity and mortality, which are increased in older patients and those with select co-morbidities. Several articles have summarized the relationship between COVID-19 and various systemic diseases in the human body, such as cardiovascular diseases, kidney disease, and digestive tract diseases. However, the association with neurodegenerative diseases is incompletely elucidated. Therefore, the aim of this article is to review the relationship between COVID-19 and neurodegenerative diseases from the perspective of ACE2, which is an important member of the pathogenesis of the diseases, thereby providing recommendations for the protection of elderly patients with neurodegenerative diseases during the COVID-19 pandemic.

### The relationship between COVID-19 and ACE2

The renin-angiotensin system (RAS) contain the classical angiotensin-converting enzyme/angiotensin II/angiotensin type 1 receptor (ACE/Ang II/AT1R) pathway and the counter-regulatory ACE2/Ang-(1-7)/Mas receptor pathway. The two pathways are important in the maintenance of homeostasis in the human body, and their imbalances could contribute to various diseases, such as kidney disease, hypertension, and cardiovascular disease [4, 5]. ACE2 is a key component of, but does not activate, the ACE2/Ang-(1-7)/Mas receptor pathway of the RAS. It is widely expressed in human tissues, such as lung, kidney, small intestine, and heart [6, 7]. There is an association between smoking and ACE2 expression, although deeper connections still need to be explored [8, 9]. Like SARS-CoV, SARS-CoV-2 uses ACE2 as its receptor for infecting human respiratory epithelial cells [10]. The SARS-CoV-2 virus coat expresses the S (Spike) protein with a receptor-binding region that directly binds to the extracellular domain of ACE2 [11]. A recent study demonstrated that the affinity of the S protein of SARS-CoV-2 to human ACE2 is even higher than that of SARS-CoV [12]. Because SARS-CoV-2 need to bind to the ACE2 receptor before entering the host cells, the distribution and expression of ACE2 may be critical for the target organ of the SARS-CoV-2 infection [10].

Several lines of evidence suggest that the binding of SARS-CoV-2 to the ACE2 receptor leads to ACE2 depletion. This inhibits the ACE2/Ang (1-7)/Mas receptor pathway and disrupts the balance of the RAS system. The result is aggravation of severe acute pneumonia and cardiovascular complications, such as myocardial injury, myocarditis, acute myocardial infarction, heart failure, and arrhythmia in patients with COVID-19 [13, 14]. ACE2 plays a critical role in the course of COVID-19. It is not only a receptor but is also involved in post-infection regulation, including immune response, cytokine secretion, and viral genome replication [15]. Therefore, the target organs, which are prone to experience COVID-19 complications, have some consistency in the distribution and expression levels of the ACE2 receptor.

**Figure 1:**
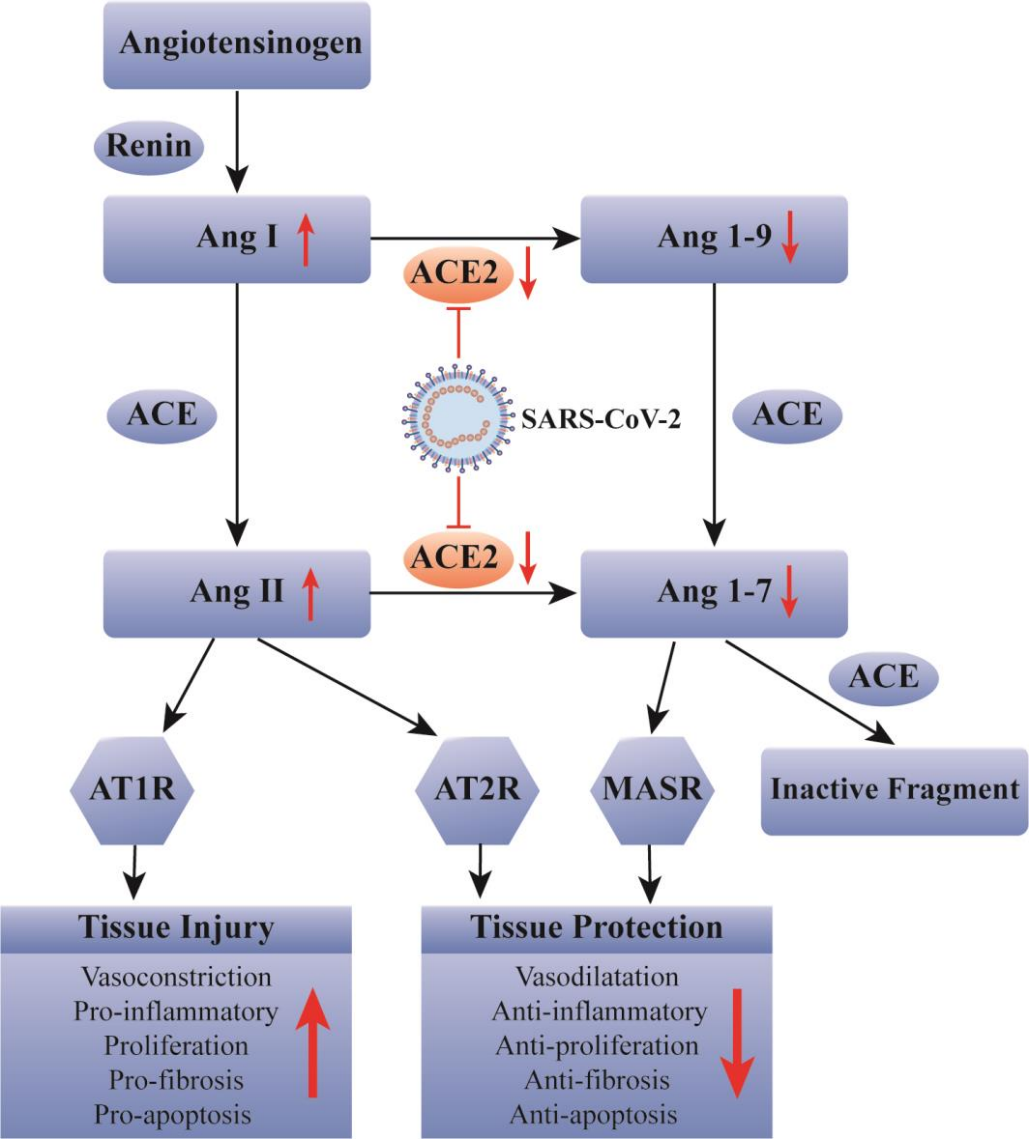
**SARS-CoV-2 affects the renin-angiotensin system via ACE2.** ACE: angiotensin converting enzyme; Ang: angiotensin; AT1R: Ang II type 1 receptor; AT2R: Ang II type 2 receptor; MASR: MAS receptor.

It has been well recognized that ACE2 plays a key role in SARS-CoV-2 infection. Therefore, some researchers have suggested that it is a potential therapeutic target for COVID-19. Recently, scientists from Spain, Sweden, and Canada, proposed to use recombinant human ACE2 (rhACE2) to “neutralize” COVID-19 to prevent it from entering human cells and causing infection. This new idea for the prevention of SARS-CoV-2 infection focused on the early stage of viral infection, whereas this is theoretical and has not been validated in human [16]. In summary, potential COVID-19 therapies need to be further explored, and whether blocking ACE2 is a feasible way to relieve COVID-19 remains to be determined (Figure 1).

### Relationship between ACE2 and neurodegenerative diseases

Neurodegenerative diseases are caused by the loss of neurons or their myelin sheaths. These diseases are common in the elderly and include Alzheimer’s disease (AD), Parkinson’s disease (PD), Huntington’s disease, and multiple sclerosis (MS). The pathological manifestations of AD include abnormal accumulation of amyloid-β (Aβ) in the brain and aggregation of hyperphosphorylated tau-like proteins in neurons to form nerve fiber tangles [17]. The typical PD features are the pervasive presence of alpha-synuclein-positive Lewy bodies, loss of dopamine neurons, and dystrophic Lewy neurites [18].

RAS also plays an indispensable role in brain function, and studies have demonstrated that renin, ACE, Ang II, and Ang (1-7) are all found in the central nervous system. They participate in the blood pressure regulation, water and food intake, maintenance of the blood–brain barrier, and even movement, learning, memory, and emotional control [19]. Genetics, epidemiology, and clinical data all indicate that overactivation of the RAS system is one of the major elements in the pathogenesis of neurodegenerative diseases [19]. Furthermore, ACE2, including the ACE2/Ang (1-7)/Mas axis, plays a regulatory role in neurodegenerative diseases [20]. RAS dysregulation is related to the pathogenesis of AD, and drugs targeting the RAS can improve cognitive impairment and AD [21–23]. Additionally, the level of ACE was decreased in the cerebrospinal fluid of AD patients, and it was negatively correlated with the level of Aβ [24, 25]. Moreover, electron microscopy revealed that ACE could delay fiber formation in a dose-dependent manner and reduce susceptibility to AD [26]. The amyloid deposited in the brain of AD patients mainly refers to the longer and neurotoxic Aβ42 and Aβ43 [27]. ACE, a neuroprotective factor, can transform Aβ42 to the shorter and less neurotoxic Aβ40 in the brain of amyloid precursor protein transgenic mice. ACE2, the homologue of ACE, functions as a powerful negative regulator in the RAS, balancing the multiple functions of ACE. Similarly, ACE2 can convert Aβ43 to Aβ42, and the combined use of ACE and ACE2 can convert Aβ43 to Aβ40 [27]. In a preclinical model experiment of AD, ACE2 was shown to prevent and reverse the pathological changes and cognitive impairment associated with amyloid in the hippocampus [28]. PI3K/Akt, an intracellular signaling pathway, can be regulated by the activation of the ACE2/Ang(1-7)/Mas axis, which can exert neuroprotective, anti-apoptotic, anti-inflammatory, and neurotrophic effects and can inhibit cognitive deficits in AD [29]. Most importantly, ACE2 was found in the brain tissue of AD patients after death. It was mainly distributed in the endothelial cells and smooth muscle cells of cerebral arteries, but ACE2 activity was reduced, and the ACE2/Ang (1-7)/Mas axis was imbalanced. Collectively, these findings further confirmed that therapeutic strategies activating ACE2 signaling has a potential to exert protective effects for AD [30].

**Figure 2:**
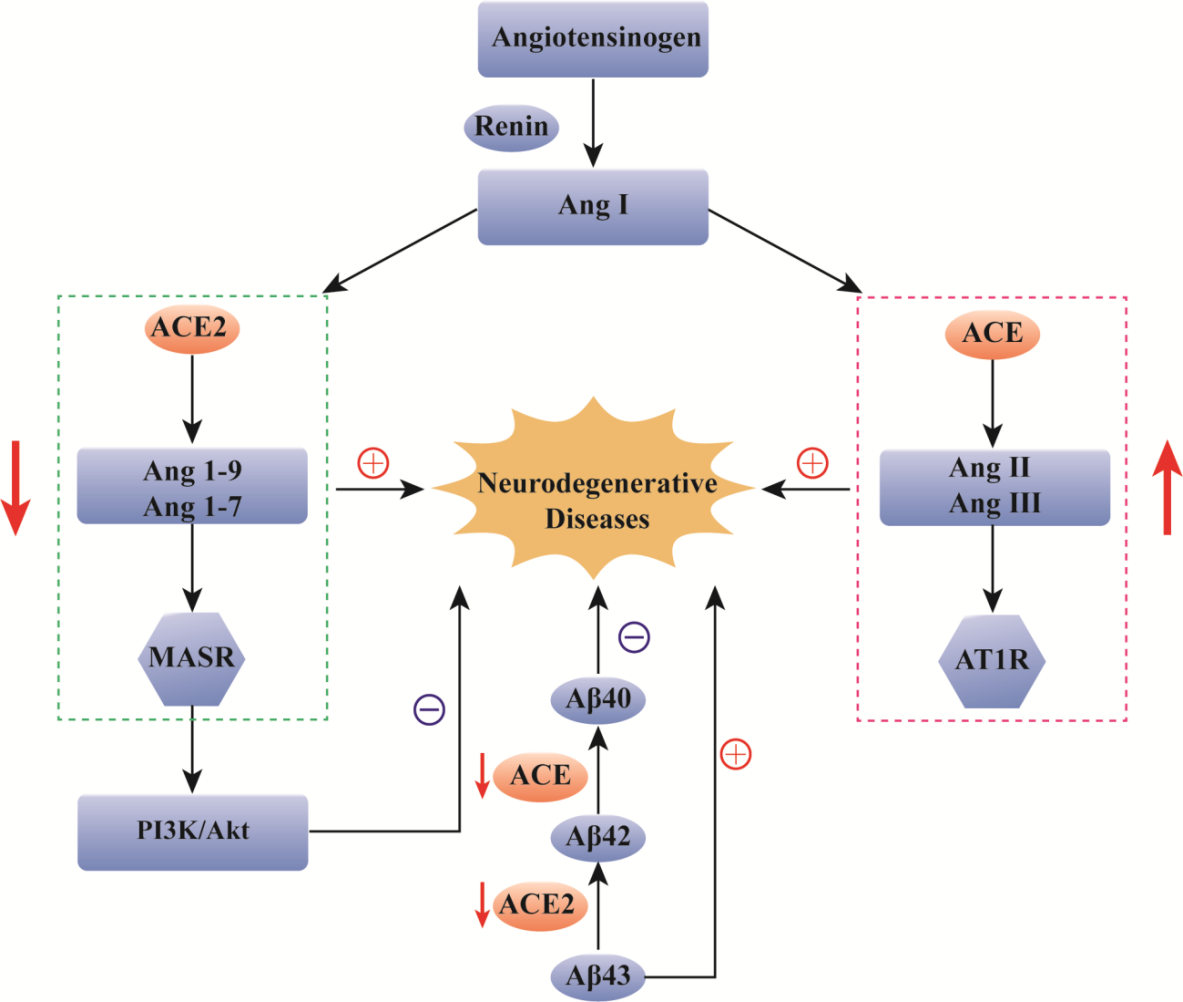
**The role of ACE2 in neurodegenerative diseases.** Reduction of ACE2 and ACE levels has been found in many neurodegenerative diseases, such as AD, PD and MS. The over-activation of ACE/Ang2/AT1 pathway and the imbalance of ACE2/Ang(1-7)/Mas pathway have been found to be closely related to the occurrence of AD. In addition, ACE2/Ang(1-7)/Mas pathway can alleviate the symptoms of AD by regulating the PI3K/Akt signaling pathway. ACE and ACE2 are also involved in the transformation of Aβ protein in AD patients. ACE: angiotensin converting enzyme; Ang: angiotensin; AT1R: Ang II type 1 receptor; Aβ: amyloid-β; MASR: MAS receptor.

ACE and ACE2 have also been detected in the cerebrospinal fluid of patients with PD and MS. Zubenko et al. detected a decrease in the ACE level in the cerebrospinal fluid of PD patients [31], and that Kawajiri et al. detected a decrease in the levels of ACE and ACE2 in the cerebrospinal fluid of MS patients [32]. However, there are few studies on the role of ACE2 in the pathological processes of PD and MS, and the specific mechanism needs to be explored (Figure 2).

### Neurodegenerative diseases and the elderly

In 2013, the US official statistics recorded 84,767 deaths due to AD which is the 5^th^ leading cause of death for Americans over 65. In 2016, an estimated 700,000 Americans over the age of 65 died of AD [33]. In 2015, the number of patients with PD is 6.9 million worldwide, and it is expected to reach 14.2 million by 2040 with a strong age dependence [34]. Various pathological changes occur in neurodegenerative diseases, such as oxidative stress, immune inflammation, neurotoxicity, and mitochondrial dysfunction [35–37]. However, the main risk factor is still advanced age [38]. In addition, both hereditary and non-hereditary neurodegenerative diseases are similar in the age of onset and are part of the aging process [39]. Advanced age is often accompanied by a gradual decline in physiological function, and the pathophysiological changes related to old age also increase the risk of neurodegenerative diseases [40]. Elderly individuals often have chronic diseases, such as hypertension, and atherosclerosis has become a common phenomenon. Unfortunately, atherosclerosis and the accumulation of hypoperfusion-related vascular factors increase the risk of AD in the elderly [41]. The neural regeneration ability of the human brain declines rapidly with age [42], accompanied by cognitive impairment, which causes neurodegenerative diseases, such as AD and PD [43]. Dopamine systems in both human and rodent brains exhibit age-related presynaptic and postsynaptic decline. Even without neurological disease, the number of dopamine neurons and dopamine levels of middle-aged individuals will have declined by 50% by the time they are elderly [43]. A previous study reported that neural stem cells (NSCs) were derived from the SVZ on postnatal 7 d, 1 m, and 12 m-old mice. Quantitative proteomic analysis of age-related subventricular zone proteins in mice revealed that the proliferative capacity of neural stem cells decreased with age [44].

### The elderly and COVID-19

During the COVID-19 pandemic, the elderly have been at increased risk for morbidity and mortality, and it is becoming a huge challenge for the global health system [45, 46]. The Centers for Disease Control and Prevention has reported that although people over 65 years old account for 17% of the population of the United States, they account for 31% of COVID-19 infections, 45% of hospitalizations, 53% of intensive care unit admissions, and 80% of deaths [47]. In China, even when public health measures are strictly enforced, people over the age of 60 account for 80% of deaths [48]. The elderly living in communities, nursing homes, assisted living facilities, and other communal living environments were reported to be at a high risk [49]. To reduce the mortality, UK has proposed the isolation of people over 70 years old, whereas the WHO has recommended a threshold of 60 years old. Some people have even called for elderly medical workers to avoid to be the epidemic prevention frontline workers [50]. Laboratory and imaging examinations have shown that the proportion of increased leukocytes and neutrophils, the proportion of decreased lymphocytes, and the incidence of multi-lobular lesions of the lungs in elderly patients were all higher than those in middle-aged patients [50]. A study revealed that in Wuhan, comorbidities, duration from paroxysm to hospitalization, renal dysfunction, and elevated procalcitonin levels were significantly associated with higher death rates in the elderly (>65 years) [51].

The symptoms of COVID-19 in the elderly are often atypical such as delirium, low-grade fever, and abdominal pain [52, 53], which undoubtedly hinders early diagnosis and timely treatment and prolongs the onset of hospitalization. A survey of 21 patients with an average age of 70 years in Washington indicated that SARS-CoV-2 infection resulted in various forms of organ damage, including acute respiratory distress syndrome (71%), acute kidney injury (20%), heart injury (33%), and liver dysfunction (15%) [54]. In addition, the elderly experience age-related immunosenescence, such as thymus degeneration and bone marrow dysfunction. These affect the cellular and molecular elements of innate and adaptive immunity, thus weakening the immune system. Consequently, elderly individuals are easily infected and vulnerable to death due to comorbid diseases [55]. Clinical data reveal that the average number of days from the first symptom to death for people over 70 years old (about 11.5 days) is shorter than that of younger people (20 days), suggesting that the disease progresses more rapidly in elderly individuals [56]. Regardless of environmental or physiological conditions, the elderly are more easily infected with COVID-19 and are more likely to die.

### Protective measures

ACE2, which is associated with improvements in neurodegenerative diseases, is the viral receptor that facilitates the entry of the virus into human cells. Thus, during this pandemic, the elderly with neurodegenerative diseases are at risk, and strengthening the attention and protection of these patients is important. However, since there is no vaccine or specific antiviral drug for COVID-19, the best way is to avoid exposure to the virus. Elderly people with a neurodegenerative disease should be advised to self-isolate at home. To protect the elderly and thus relieve overburdened health systems, countries around the world have begun to implement blockades and quarantine measures to reduce the spread of SARS-CoV-2.
